# Superlattice Microstructured Optical Fiber

**DOI:** 10.3390/ma7064567

**Published:** 2014-06-16

**Authors:** Ming-Leung Vincent Tse, Zhengyong Liu, Lok-Hin Cho, Chao Lu, Ping-Kong Alex Wai, Hwa-Yaw Tam

**Affiliations:** 1 Photonics Research Centre, Department of Electrical Engineering, The Hong Kong Polytechnic University, Hung Hom, Kowloon, Hong Kong; E-Mails: zhengyong.liu@connect.polyu.hk (Z.L.); hwa-yaw.tam@polyu.edu.hk (H.-Y.T.); 2 Photonics Research Centre, Department of Electronic and Information Engineering, The Hong Kong Polytechnic University, Hung Hom, Kowloon, Hong Kong; E-Mails: stephen.cho@connect.polyu.hk (L.-H.C.); chao.lu@polyu.edu.hk (C.L.); alex.wai@polyu.edu.hk (P.-K.A.W.)

**Keywords:** superlattice, birefringent, photonic crystal fiber, sensing, microstructured fiber

## Abstract

A generic three-stage stack-and-draw method is demonstrated for the fabrication of complex-microstructured optical fibers. We report the fabrication and characterization of a silica superlattice microstructured fiber with more than 800 rhomboidally arranged air-holes. A polarization-maintaining fiber with a birefringence of 8.5 × 10^−4^ is demonstrated. The birefringent property of the fiber is found to be highly insensitive to external environmental effects, such as pressure.

## 1. Introduction

Photonic crystal fiber (PCF), also known as microstructured optical ﬁbers (MOFs) or “holey” fiber, produce their light guidance effect through a patterning of tiny holes, which run along the entire length of the fiber. In the simplest case, where the core of the fiber is solid, and surrounded by many air channels, and thus lower its effective refractive index, and allowing guidance by total internal reﬂection. They can therefore be made using a single material, without chemical doping. Since the first photonic crystal fiber was reported in 1996 [1], researchers had been wanted to study PCF designs with non-circular holes. Many theoretical studies were unpublished based on unrealistic fabrication targets. Elliptical, rhomboidal and square hole shapes were studied in the few published works [2,3,4]. The combined effects of surface tension and hole pressure during fiber drawing, make it very difficult to control the shape of the holes in the fiber structure. Finding a balance is one of the main problems in producing PCFs with non-circular holes. Recently, the concept of a superlattice photonic crystal fiber was reported [5]. It is a novel and practical approach for the fabrication of PCFs with complex structures [6]. Any arbitrary shaped holes can be realized by arranging many smaller holes to approximate the designed shape. As an example, a high birefringence fiber design was proposed and the birefringent property was investigated. The idea was to design a PCF with hexagonal lattice and effective-elliptical-holes in the cladding; within each effective super-structured cell, only constant diameter circular holes are used. This is an alternative design concept to achieve high birefringence in a PCF other than by employing bow-tie-like [7], elliptical holes [8], squeezed elliptical core and holes [9] or elliptical core [10] structures.

In this paper, a comparison of the birefringence for three different PCFs with different structure under external pressure is made. We report the fabrication of the first proof-of-concept silica superlattice PCF. The proposed all-silica high birefringent superlattice photonic crystal fiber (SL-PCF) can be fabricated by the structured-element-stacking method [11]. However, here, the preforms were stacked using circular silica capillaries and circular structured elements only. The focus of the work was on the fabrication method and the pressure response affected by the makeup of different fiber structures, especially by a more complex structure that of the superlattice reporting here. Together with the modified-stack-and-draw techniques demonstrated in [12], the authors intend to provide a complete practical solution for fabrication of complex PCFs; investigating the limit of the stack-and-draw techniques manually performed for the past two decades. The multi-stage stack-and-draw method leads to the possibility of realizing designs with multiple structures, multiple hole shapes and/or multiple cores within a single PCF. Thus, many studies have been carried out for novel fiber designs that may lead to new optical phenomena and applications.

The mechanical properties derived from the microstructure of PCFs have not been studied in detail in the past. The mechanical effect on the optical property of a PCF is an interesting area in engineering, especially for sensing applications: an example of a novel lateral load insensitive design utilized this property is proposed in [13]. Here, the SL-PCF presented has a birefringence of 8.5 × 10^−4^, which is comparable with that of commercially available polarization-maintaining conventional and photonic crystal fibers. It was found that the birefringence was highly insensitive to external pressure.

## 2. Fabrication

**Figure 1:**
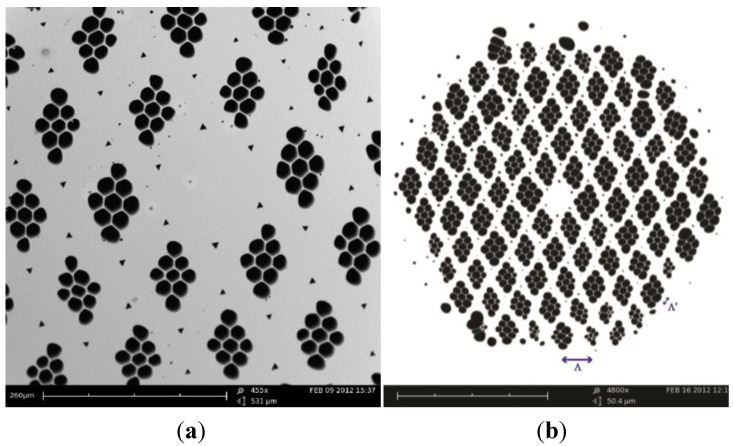
SEM images of (**a**) the central region of the final cane; and (**b**) the SL-PM-PCF. Scale bar: 260 µm and 20 µm respectively

**Figure 2:**
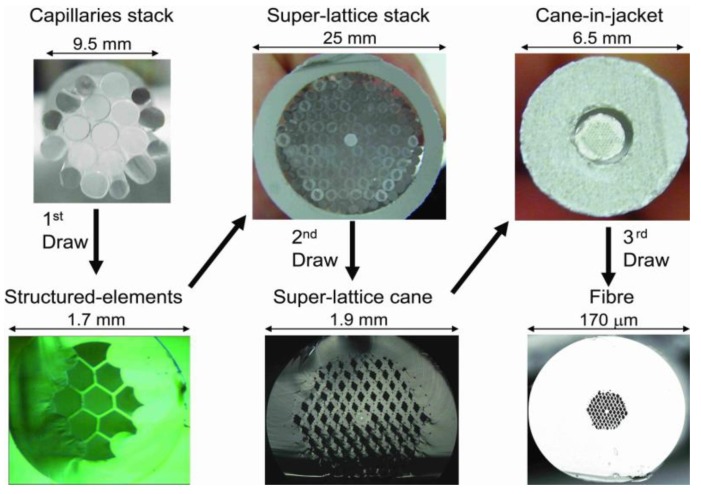
Photos of stacks and canes to illustrate the structured-element-stacking method (the illuminated Ge-doped area can be seen in the core of the superlattice cane).

The SEM images of the central region of the cane and fiber are shown in Figure 1a,b, respectively. The fiber was fabricated by a multiple stack-and-draw method. Figure 2 illustrates the fabrication scheme with photos of stacks and canes. Initially, nine capillaries with air-filling ratio of 0.95 were rhomboidally stacked together; the stack was then drawn to structured-elements with outer diameter (OD) of 1.69 mm. Next, one core element (~3% of the central area is Ge-doped with ∆n ≈ 0.55%) plus 90 structured-elements were hexagonally stacked together to form the core and five rings of rhombus-like holes in the cladding, respectively. Each structured-element was orientated manually to be aligned so that two clearly identifiable axes were formed in the overall structure. This stack was then drawn into final canes (OD = 1.9 mm). In the final step, a selected cane was inserted into a jacketing tube and drawn into the superlattice polarization maintaining photonic crystal fiber, SL-PM-PCF (OD = 170 µm). All the elements and fiber were drawn on a 5.5 m dual sided research fiber drawing tower at The Hong Kong Polytechnic University (Nextrom OFC20, Vantaa, Finland).

Note that not all the structured-elements were aligned perfectly. However, only an overall averaged alignment was required to achieve high birefringence. The imperfection clearly shows the limitation of the stacking technique when carried out manually by simple eye inspection. A better result may be possible if the stacking is done robotically, or by using non-circular structured-elements.

## 3. Simulations

**Figure 3:**
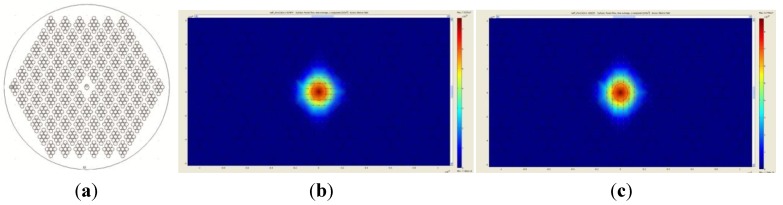
(**a**) The ideal structure used in the simulation, where Ʌ = 4 µm, Ʌ′ = 1.1 µm and d′ = 1 µm (with Ge-doped area indicated at the center); (**b**) *x*-polarized mode, fast axis effective index = 1.427874; (**c**) *y*-polarized mode, slow axis effective index = 1.428225 of the simulated mode profile.

We employed a full-vector finite-element method (FEM) and anisotropic perfectly matched layers using the simulation software Multiphysics (COMSOL AB, Stockholm, Sweden), to investigate the guided modes of the SL-PCF. An ideal structure was used, where the lattice-to-lattice pitch, Ʌ = 4 µm, hole-to-hole pitch within a structured-element cell, Ʌ′ = 1.1 µm and hole diameter d′ = 1 µm, see Figure 3a. The simulated fiber was found to be single mode at 1550 nm. The simulated effective indices for the fast and slow axes were 1.427874 and 1.428225, respectively. Therefore, the simulated birefringence was 3.51 × 10^−4^, which agreed fairly well with the experimental result. It is worth noting that, we also simulated the same structure without the Ge-doped region, and the resultant birefringence was 3.89 × 10^−4^. Therefore, the low concentration of Ge in the core only contributes slightly to the birefringence. Germanium is included in the fiber core to facilitate future UV inscription of Bragg gratings. The simulated mode profile is shown in Figure 3b,c.

## 4. Birefringence and Pressure Measurements

**Figure 4:**
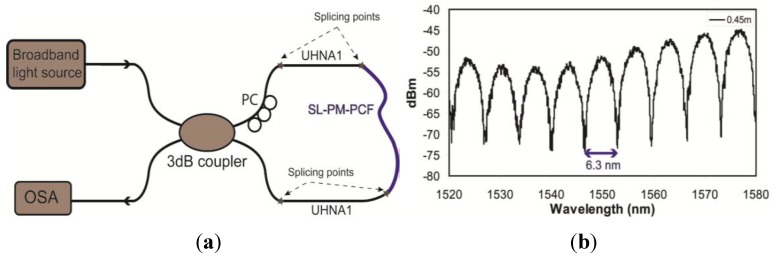
(**a**) Configuration of Sagnac loop for birefringence measurement; (**b**) output optical spectrum of the Sagnac loop.

The birefringence of the SL-PCF was measured using the Sagnac loop interferometer method [14]. The experimental setup is shown in Figure 4a. The Sagnac loop consisted of a standard 3dB fiber optic coupler, two pieces of conventional high NA fiber (Nufern-UHNA1) spliced to the two ports of the coupler on one side. A piece of SL-PCF with a length of 0.45 m was spliced between the two UHNA1 fibers. The total loss of the two PCF-UHNA1 splicing points was <1.4 dB [15]. The coupler splits the input signal equally into two signals with a π/2 phase difference between them. The two signals counter-propagate through the SL-PCF before they interfere again at the coupler. A broadband superbright-light-emitting-diode source was used at the input, and the output signal was observed using an optical spectrum analyzer. The transmission spectrum was approximately a periodic function of the wavelength, and the birefringence, *B*, can be estimated by

*B* = λ^2^∕(δλ*·L*) (1)

where λ is the center wavelength; δλ is the period of the interference pattern; and *L* is the length of the SL-PCF. It is assumed that the effect of temperature is minimal for silica PCF, which has a very low thermal expansion coefficient [14]. Figure 4b shows the output optical spectrum at the 1550 nm wavelength band. The spacing between two adjacent transmission minima is 6.3 nm, which correspond to a birefringence of 8.5 × 10^−4^ at 1550 nm. The birefringence of the real fiber is higher than the ideal structure because of the core element is also being slightly elliptical, see Figure 1. This is similar to the enhanced birefringence observed with the squeezed elliptical core and holes PCF reported in [9].

A pressure response experiment was also carried out using a setup with Sagnac loop interferometer and a high pressure oil tank [16]. A piece of the SL-PM-PCF with length of ~30 cm was used in the experiment. In order to obtained fibers with smaller outer diameter, hydrofluoric acid was used to etch the outer silica region of the SL-PM-PCF, while keeping the structural region unchanged. The post-etched diameter was 110 µm. The experiment was carried out at a wavelength of 850 nm, and the fiber was found to be effectively single mode; higher order guiding modes of greater losses lead to lower visibility resonation within the Sagnac loop. The pressure response is expected to be about three times higher than that at 1550 nm. The main benefit of working at 850 nm is that a low-cost CCD interrogator can be used, which also provide a much higher spectrum scanning speed than that of a standard optical spectrum analyzer [16].

It was found that the pressure response was ~1.6 nm/MPa at 850 nm. This is consistent with the simulated value of 0.5 nm/MPa at 1550 nm for an ideal structure using a FEM tool. Next, the result was compared to that found by using commercially available PM-PCF (NKT Photonics PM-1550-01). A piece of this fiber with the original outer diameter of 125 µm was used. It was found that the pressure response was ~9.7 nm/MPa at 850 nm, which is again fairly consistent with the simulated value of 3.8 nm/MPa at 1550 nm. Therefore, a pressure response difference of more than six times between the two fibers is observed. Finally, a suspended core fiber was used to perform the same experiment [17]. It had an outer diameter of 125 μm, with six wedge-shaped holes; each had a dimension of ~22 μm by ~27 μm. The thickness of the struts was ~730 nm. The suspended elliptical core had a major and minor axis of ~8 μm × 4 μm, with a birefringence of ~4.8 × 10^−4^ at 1550 nm. The pressure response was ~2.8 nm/MPa at 1550 nm. Again, much higher than that of the superlattice fiber.

It can be concluded that for the PM-1550-01 and superlattice fibers, which have holey structured region diameter of ~40 µm, similar outer diameter and birefringence of ~8 × 10^−4^, exhibit very different pressure responses. The suspended core fiber representing another very different structural makeup and the response to external pressure is also different. The pressure induced birefringence response can vary greatly according to the complexity of the cladding microstructures. The birefringence of the superlattice PCF is almost independent to external pressure, which may provide a stable operation in harsh environmental conditions such as the ones under high pressure downhole [18].

## 5. Conclusions

We have shown experimentally that a high birefringent superlattice microstructured fiber can be fabricated using the proposed structured-element-stacking method. The initial circular capillaries can be arranged to form rhombus-like super-cell holes, and can be manually aligned on average over the entire structure with good birefringence result. The measured birefringence matched well with the simulated ideal structure. The fiber exhibited very low pressure (and temperature) response in comparison to other PCF microstructures. A much higher birefringence is expected for a SL-PCF with smaller core and pitch [5]. It is expected that this work will further widen the structured optical fiber research field, especially in the mechanical area.
